# Bidirectional association between asthma and chronic rhinosinusitis: Two longitudinal follow-up studies using a national sample cohort

**DOI:** 10.1038/s41598-020-66479-8

**Published:** 2020-06-12

**Authors:** Gwanghui Ryu, Chanyang Min, Bumjung Park, Hyo Geun Choi, Ji-Hun Mo

**Affiliations:** 1 Department of Otorhinolaryngology-Head and Neck Surgery, Soonchunhyang University College of Medicine, Cheonan, Republic of Korea; 2 Hallym Data Science Laboratory, Hallym University College of Medicine, Anyang, Republic of Korea; 3 Graduate School of Public Health, Seoul National University, Seoul, Republic of Korea; 4 Department of Otorhinolaryngology-Head & Neck Surgery, Hallym University College of Medicine, Anyang, Republic of Korea; 5 Department of Otorhinolaryngology, Dankook University College of Medicine, Cheonan, Republic of Korea; 6 Beckman Laser Institute Korea, Dankook University College of Medicine, Cheonan, Republic of Korea

**Keywords:** Asthma, Epidemiology, Chronic inflammation

## Abstract

The aim of this study was to evaluate an epidemiologic association of asthma and chronic rhinosinusitis (CRS) using a national sample cohort of the Korean population. We collected data from the Korean Health Insurance Review and Assessment Service-National Sample Cohort between 2002 and 2013, and two different case-control cohorts were designed (1st: asthmatic patients matched in a 1:1 ratio with 204,119 non-asthmatics as control I, 2nd: CRS patients matched in a 1:4 ratio with 124,020 non-CRS patients as control II). Bidirectional association was examined using Cox proportional hazard models stratified by age, sex, income, and region of residence. Patients with asthma had an increased risk of developing CRS [adjusted hazard ratio (95% confidence interval) = 1.74 (1.67–1.80)], both with nasal polyps [1.55 (1.36–1.78)], without nasal polyps [1.74 (1.67–1.81)]. In the second cohort, patients with CRS had increased risk of developing asthma [1.85 (1.80–1.91)] with similar results for those with and without nasal polyps. The strongest association for risk of CRS was in 20–39 years old men with asthma [2.41 (1.97–2.96)], while the strongest association for increased risk of asthma in those with CRS group was also seen in this same subgroup [2.40 (2.18–2.63)]. CRS and asthma had a bidirectional influence on each other. CRS increased the risk of asthma, and asthma increased the risk of CRS, especially in young men.

## Introduction

Chronic rhinosinusitis (CRS) is a common upper airway disorder and a significant health problem that poses a large economic burden on society with increasing frequency^1–3^. Diagnostic criteria of CRS are as follow; an inflammation of the nose and the paranasal sinus lasting for more than 12 weeks which is characterized by nasal symptoms such as blockage, discharge, facial pain, and loss of smell^4^. CRS is divided into two phenotypes based on the presence of nasal polyps: CRS with nasal polyp (CRSwNP) and CRS without nasal polyp (CRSsNP)^4^. The dichotomous phenotypes revealed different clinical manifestation and molecular endotype^5,6^. The etiology of CRS, including CRSwNP and CRSsNP, is still not well understood and has been studied extensively. Several hypotheses have been proposed to elucidate the pathophysiology of CRS, including fungal, immune barrier, superantigen, and biofilm hypotheses as well as the suspicion of defects in the eicosanoid pathway^7^.

The prevalence of asthma is increased in patients with CRS, and this strong association between asthma and CRS has been widely noted. The prevalence of asthma in patients with CRS has been reported in a wide range from 4 to 44%^8–14^. The comorbidity rate of asthma in CRSwNP patients is much higher than that in CRSsNP patients^5^. About 20 to 60% of CRSwNP patients had comorbid asthma in Europe^15,16^. In asthma patients, 41–51% had CRS in a large cohort study in United States^17^. However, the interaction between the two conditions is not clearly understood, and a recent study suggests that increased periostin levels may predispose patients with CRSwNP to comorbid asthma^18^. Since the conception of “one airway disease” or “unified airway disease” was introduced, epidemiological correlation among asthma, CRS, and allergic rhinitis have been studied^19,20^. Also, immune-pathological mechanisms shared by both CRS and asthma have been identified, especially tissue remodeling, including epithelial hyperplasia, goblet cell hyperplasia, basement membrane thickening, and increased matrix deposition and plasma proteins^21^. Clinically, bidirectional effects of provocation tests were observed such that nasal provocation tests induced bronchial inflammation and bronchial stimulation induced nasal inflammation^22,23^.

Although the relation between the CRS and asthma has been studied in various ways, most of those studies were based on small cohorts or small population studies. However, the association of asthma and CRS in a large epidemiologic study has not been investigated. In this study, we investigated the bidirectional association between CRS and asthma using a national sample cohort.

## Results

### Study I: Asthma increases the risk of developing CRS

**Figure 1:**
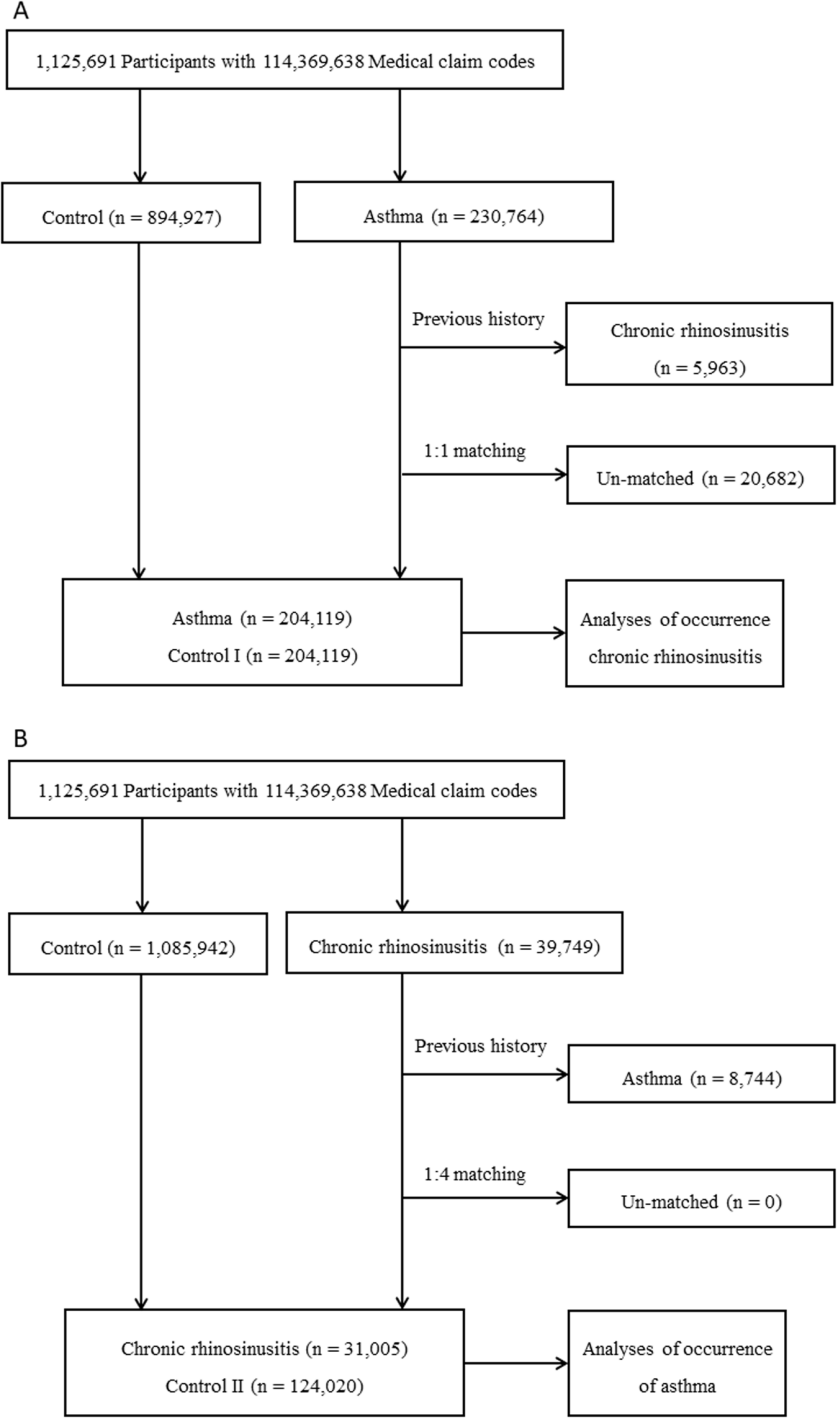
(**A**) Schematic illustration of the participant selection process that was used in the present study. Of a total 1,125,691 participants, 204,119 asthma patients were matched with 204,119 control I participants for age, group, sex, income group, and region of residence. (**B**) Schematic illustration of the participant selection process that was used in the present study. Of a total of 1,125,691 participants, 31,005 CRS patients were matched with 124,020 control II participants for age, group, sex, income group, and region of residence.

**Figure 2:**
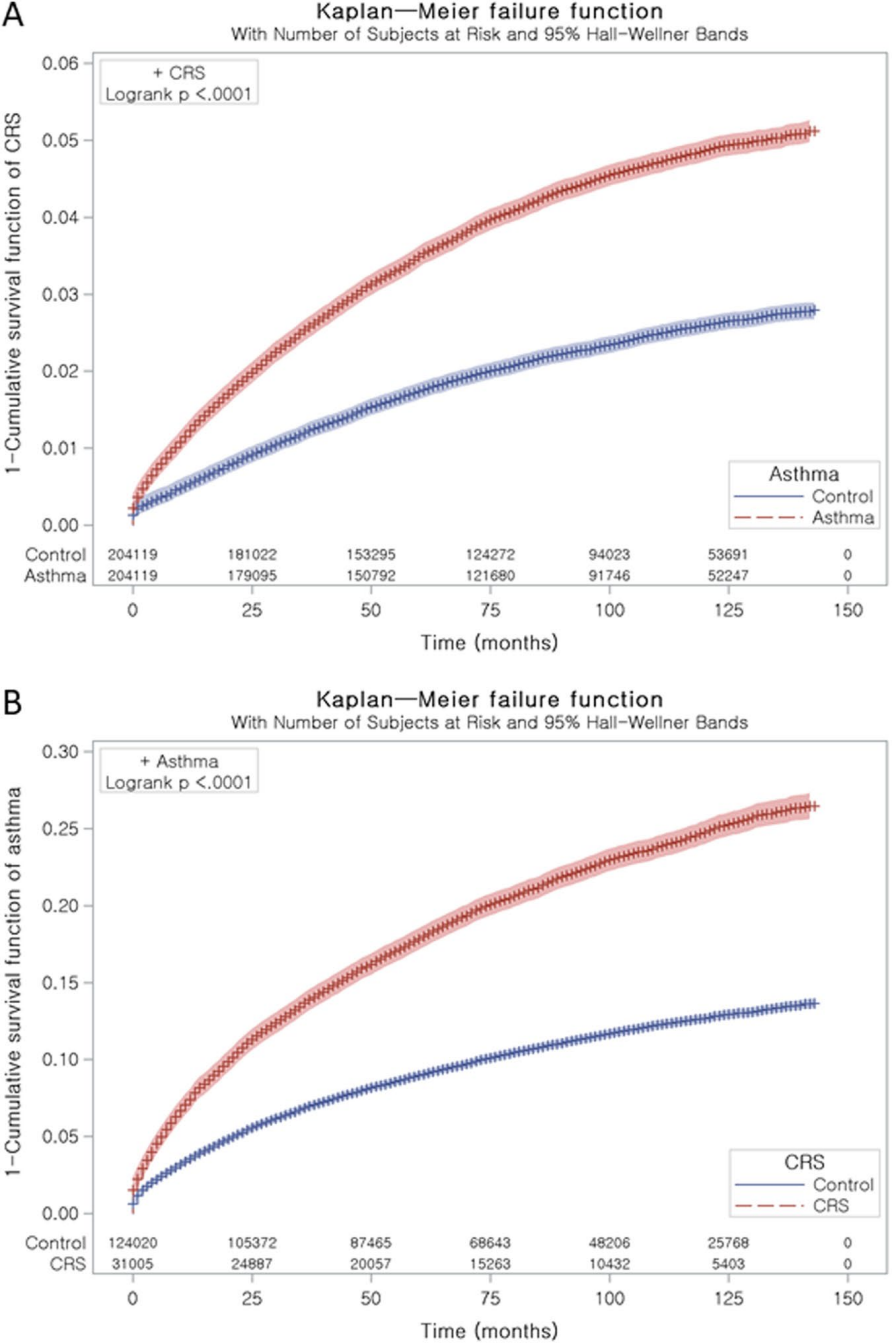
(**A**) Kaplan-Meier survival analysis of CRS in both asthma and control I. (**B**) Kaplan-Meier survival analysis of asthma in both CRS and control II.

Asthma patients were 1:1 matched with non-asthmatic patients (control I) who had same general characteristics (Fig. 1A). The average time from index date, the date of the diagnosis of asthma, to CRS diagnosis was 35.2 months (standard deviation, SD = 31.4) in the asthma group and 39.5 months (SD = 33.4) in the control I group (Fig. 2A). The rate of developing CRS was higher in the asthma group [3.9% (8,043/204,119)] than in the control I group [2.1% (4,185/204,119), P < 0.001] (Table 1). The characteristics (e.g., age, sex, income, and region of residence) of two groups were exactly the same due to the matching (*P* = 1.000), while the rates of comorbid atopic dermatitis, chronic obstructive pulmonary disease (COPD), and Charlson comorbidity index (CCI) score ≥ 2 were higher in the asthma group compared to the control I (each *P* < 0.001).

**Table 1:** General characteristics of participants.

Characteristics	Study I			Study II		
	Asthma (n, %)	Control I (n, %)	P-value	CRS (n, %)	Control II (n, %)	P-value
Age (years old)			1.000			1.000
0–4	60,672 (29.7)	60,672 (29.7)		2,145 (6.9)	8,580 (6.9)	
5–9	17,273 (8.5)	17,273 (8.5)		1,979 (6.4)	7,916 (6.4)	
10–14	7,432 (3.6)	7,432 (3.6)		1,746 (5.6)	6,984 (5.6)	
15–19	4,266 (2.1)	4,266 (2.1)		1,642 (5.3)	6,568 (5.3)	
20–24	4,429 (2.2)	4,429 (2.2)		1,480 (4.8)	5,920 (4.8)	
25–29	7,158 (3.5)	7,158 (3.5)		1,950 (6.3)	7,800 (6.3)	
30–34	10,639 (5.2)	10,639 (5.2)		2,418 (7.8)	9,672 (7.8)	
35–39	10,776 (5.3)	10,776 (5.3)		2,523 (8.1)	10,092 (8.1)	
40–44	10,575 (5.4)	10,575 (5.4)		2,500 (8.1)	10,000 (8.1)	
45–49	11,104 (5.3)	11,104 (5.3)		2,637 (8.5)	10,548 (8.5)	
50–54	10,866 (5.1)	10,866 (5.1)		2,540 (8.2)	10,160 (8.2)	
55–59	10,345 (5.1)	10,345 (5.1)		2,270 (7.3)	9,080 (7.3)	
60–64	10,415 (5.1)	10,415 (5.1)		1,831 (5.9)	7,324 (5.9)	
65–69	10,411 (5.1)	10,411 (5.1)		1,555 (5.0)	6,220 (5.0)	
70–74	8,271 (4.1)	8,271 (4.1)		1,024 (3.3)	4,096 (3.3)	
75–79	5,278 (2.6)	5,278 (2.6)		503 (1.6)	2,012 (1.6)	
80–84	2,890 (1.4)	2,890 (1.4)		187 (0.6)	748 (0.6)	
85+	1,319 (0.6)	1,319 (0.6)		75 (0.2)	300 (0.2)	
Sex			1.000			1.000
Male	87,638 (42.9)	87,638 (42.9)		16,019 (51.7)	64,076 (51.7)	
Female	116,481 (57.1)	116,481 (57.1)		14,986 (48.3)	59,944 (48.3)	
Income			1.000			1.000
1 (lowest)	26465 (13.0)	26465 (13.0)		3,859 (12.4)	15,436 (12.4)	
2	28,306 (13.9)	28,306 (13.9)		4,065 (13.1)	16,260 (13.1)	
3	40,593 (19.9)	40,593 (19.9)		5,501 (17.7)	22,004 (17.7)	
4	54,289 (26.6)	54,289 (26.6)		7,624 (24.6)	30,496 (24.6)	
5 (highest)	54,466 (26.7)	54,466 (26.7)		9,956 (32.1)	39,824 (32.1)	
Region of residence			1.000			1.000
Urban	92,570 (45.4)	92,570 (45.4)		15,088 (48.7)	60,352 (48.7)	
Rural	111,549 (54.6)	111,549 (54.6)		15,917 (51.3)	63,668 (51.3)	
Atopic dermatitis	39,839 (19.5)	26,492 (13.0)	<0.001*	3,343 (10.8)	9,087 (7.3)	<0.001*
COPD	22,764 (11.2)	5,597 (2.7)	<0.001*	2,119 (6.8)	4,091 (3.3)	<0.001*
CCI (score)^†^			<0.001*			<0.001*
0	123,035 (60.3)	140,816 (69.0)		11,882 (38.3)	78,362 (63.2)	
1	22,809 (11.2)	19,363 (9.5)		5,241 (16.9)	14,588 (11.8)	
≥2	58,275 (28.5)	43,940 (21.5)		13,882 (44.8)	31,070 (25.1)	
Asthma	204,119 (100.0)	0 (0.0)	<0.001*	6,270 (20.2)	12,731 (10.3)	<0.001*
CRS	8,043 (3.9)	4,185 (2.1)	<0.001*	31,005 (100.0)	0 (0.0)	<0.001*

*Chi-square test, Significance at P < 0.05.

^†^Charlson Comorbidity Index was calculated without pulmonary disease.

CCI, Charlson Comorbidity Index; CRS, chronic rhinosinusitis; COPD, chronic obstructive pulmonary disease.

Patients with asthma had an increased risk of developing CRS and the adjusted hazard ratio [aHR, (95% confidence interval)] was 1.74 (1.67–1.80). The aHRs of CRSwNP and CRS without nasal polyp (CRSsNP) were 1.55 (1.36–1.78) and 1.74 (1.67–1.81), respectively (Table 2). In all subjects with CRS in study I, the distribution of CRSwNP and CRSsNP was similar in both asthma patients (0.3% vs. 3.6%) and control I (0.2% and 1.9%) groups, still the prevalence of CRSwNP and CRSsNP were higher in asthma group than control I (Table S1). Between two CRS phenotypes, some characteristics were different, including age, sex, income, prevalence of atopic dermatitis, COPD, and CCI score (Table S2).

**Table 2:** Crude and adjusted hazard ratios (95% confidence interval) of asthma for chronic rhinosinusitis in study I.

Characteristics	Hazard ratios (HR)			
	Crude^†^	P-value	Adjusted^†‡^	P-value
HR for CRS				
Asthma	1.95 (1.87–2.02)	<0.001*	1.74 (1.67–1.80)	<0.001*
Control I	1.00		1.00	
HR for CRSwNP				
Asthma	1.89 (1.66–2.16)	<0.001*	1.55 (1.36–1.78)	<0.001*
Control I	1.00		1.00	
HR for CRSsNP				
Asthma	1.94 (1.87–2.02)	<0.001*	1.74 (1.67–1.81)	<0.001*
Control I	1.00		1.00	

*Cox-proportional hazard regression model, Significance at P < 0.05.

^†^Stratified model for age, sex income, and region of residence.

^‡^Adjusted model for atopic dermatitis, chronic obstructive pulmonary disease, and Charlson Comorbidity Index.

CRS, chronic rhinosinusitis, CRSsNP, chronic rhinosinusitis without nasal polyp; CRSwNP, chronic rhinosinusitis with nasal polyp.

In subgroup analyses stratified by age and sex, the risk of developing CRS was higher in the asthma group (P < 0.05, each comparison) (Table 3). The strongest associations for risk of CRS was in 20–39 years old men followed by same aged women [2.41 (1.97–2.96) and 1.88 (1.65–2.15), respectively].

**Table 3:** Subgroup analysis of crude and adjusted hazard ratios (95% confidence interval) of asthma for chronic rhinosinusitis according to age and sex in study I.

Characteristics	HR for CRS			
	Crude^†^	P-value	Adjusted^†‡^	P-value
Age <20 years old, men (n = 91,914)				
Asthma	1.58 (1.48–1.69)	<0.001*	1.50 (1.40–1.60)	<0.001*
Control I	1.00		1.00	
Age < 20 years old, women (n = 87,372)				
Asthma	1.67 (1.52–1.83)	<0.001*	1.56 (1.41–1.71)	<0.001*
Control I	1.00		1.00	
Age 20–39 years old, men (n = 21,644)				
Asthma	2.78 (2.28–3.38)	<0.001*	2.41 (1.97–2.96)	<0.001*
Control I	1.00		1.00	
Age 20–39 years old, women (n = 44,360)				
Asthma	2.17 (1.90–2.47)	<0.001*	1.88 (1.65–2.15)	<0.001*
Control I	1.00		1.00	
Age 40–59 years old, men (n = 31,272)				
Asthma	2.69 (2.34–3.10)	<0.001*	2.17 (1.87–2.51)	<0.001*
Control I	1.00		1.00	
Age 40–59 years old, women (n = 54,508)				
Asthma	2.17 (1.97–2.39)	<0.001*	1.85 (1.68–2.05)	<0.001*
Control I	1.00		1.00	
Age ≥60 years old, men (n = 30,446)				
Asthma	2.13 (1.86–2.45)	<0.001*	1.71 (1.47–1.99)	<0.001*
Control I	1.00		1.00	
Age ≥60 years old, women (n = 46,722)				
Asthma	2.31 (2.06–2.59)	<0.001*	1.93 (1.71–2.17)	<0.001*
Control I	1.00		1.00	

*Cox-proportional hazard regression model, Significance at P < 0.05.

^†^Stratified model for age, sex income, and region of residence.

^‡^Adjusted model for atopic dermatitis, chronic obstructive pulmonary disease, and Charlson Comorbidity Index.

CRS, chronic rhinosinusitis.

### Study II: CRS increased the risk of developing asthma

CRS patients were 1:4 matched with control I group who had same general characteristics (Fig. 1B). The time from index date, the date of the diagnosis of CRS, to asthma diagnosis was 32.9 months (SD = 32.1) in the CRS group and 34.1 months (SD = 32.5) in the control II group (Fig. 2B). The rate of asthma was higher in the CRS group [20.2% (6,270/31,005)) than in the control II group [10.3% (12,731/124,020), P < 0.001] (Table 1). The general characteristics of participants were exactly same (P = 1.000), while the rates of atopic dermatitis, COPD, and CCI score ≥ 2 were higher in the CRS group than in the control II group (P < 0.001, each comparison).

Patients with CRS had increased risk of developing asthma [1.85 (1.80–1.91)]. The risk of developing asthma among patients with in the CRSwNP and CRSsNP groups was 1.96 (1.79–2.15) and 1.84 (1.78–1.90), respectively, compared to the control II group (Table 4). In study II, asthma was more prevalent in CRSwNP and CRSsNP patients compared to each control group (Table S3). Between two CRS phenotypes, some characteristics were different, including age, prevalence of atopic dermatitis, COPD, and asthma (Table S4).

**Table 4:** Crude and adjusted hazard ratios (95% confidence interval) of chronic rhinosinusitis for asthma in study II.

Characteristics	Hazard ratios (HR)			
	Crude^†^	P-value	Adjusted^†‡^	P-value
HRs for Asthma (CRS, n = 155,025)				
CRS	2.13 (2.06–2.19)	<0.001*	1.85 (1.80–1.91)	<0.001*
Control II	1.00		1.00	
HRs for Asthma (CRSwNP, n = 22,705)				
CRS with polyp	2.25 (2.06–2.46)	<0.001*	1.96 (1.79–2.15)	<0.001*
Control II	1.00		1.00	
HRs for Asthma (CRSsNP, n = 132,320)				
CRS without polyp	2.11 (2.04–2.18)	<0.001*	1.84 (1.78–1.90)	<0.001*
Control II	1.00		1.00	

*Cox-proportional hazard regression model, Significance at P < 0.05.

^†^Stratified model for age, sex income, and region of residence.

^‡^Adjusted model for atopic dermatitis, chronic obstructive pulmonary disease, and Charlson Comorbidity Index.

CRS, chronic rhinosinusitis; CRSsNP, chronic rhinosinusitis without nasal polyp; CRSwNP, chronic rhinosinusitis with nasal polyp.

In subgroup analyses, the risk of developing asthma was higher in the CRS group (P < 0.05, each comparison) (Table 5). The strongest associations for risk of CRS was highest in men and women aged 20–39 years old [3.04 (2.67–3.47) and 2.40 (2.18–2.63), respectively].

**Table 5:** Subgroup analysis of crude and adjusted hazard ratios (95% confidence interval) of chronic rhinosinusitis for asthma according to age and sex in study II.

Characteristics	HRs for Asthma			
	Crude^†^	P-value	Adjusted^†‡^	P-value
Age <20 years old, men (n = 24,990)				
CRS	1.58 (1.48–1.69)	<0.001*	1.51 (1.41–1.61)	<0.001*
Control II	1.00		1.00	
Age <20 years old, women (n = 12,570)				
CRS	1.67 (1.53–1.83)	<0.001*	1.57 (1.44–1.72)	<0.001*
Control II	1.00		1.00	
Age 20–39 years old, men (n = 20,105)				
CRS	3.56 (3.14–4.04)	<0.001*	3.04 (2.67–3.47)	<0.001*
Control II	1.00		1.00	
Age 20–39 years old, women (n = 21,750)				
CRS	2.74 (2.53–3.02)	<0.001*	2.40 (2.18–2.63)	<0.001*
Control II	1.00		1.00	
Age 40–59 years old, men (n = 23,465)				
Migraine	2.71 (2.46–2.98)	<0.001*	2.14 (1.94–2.37)	<0.001*
Control II	1.00		1.00	
Age 40–59 years old, women (n = 26,270)				
CRS	2.42 (2.25–2.60)	<0.001*	2.00 (1.86–2.16)	<0.001*
Control II	1.00		1.00	
Age ≥60 years old, men (n = 11,535)				
CRS	2.18 (1.96–2.42)	<0.001*	1.67 (1.49–1.86)	<0.001*
Control II	1.00		1.00	
Age ≥60 years old, women (n = 14,340)				
CRS	1.99 (1.82–2.18)	<0.001*	1.64 (1.49–1.80)	<0.001*
Control II	1.00		1.00	

*Cox-proportional hazard regression model, Significance at P < 0.05.

^†^Stratified model for age, sex income, and region of residence.

^‡^Adjusted model for atopic dermatitis, chronic obstructive pulmonary disease, and Charlson Comorbidity Index.

CRS, chronic rhinosinusitis.

## Discussion

Many epidemiologic studies have identified the risks of CRS among asthma patients and vice versa. However, this is the first study to examine the bidirectional association of CRS and asthma using nationwide population-based data. This study demonstrated that the risk of CRS in asthma patients and that of asthma in CRS patients was higher than that of each respective control group. In the subgroup analysis, both diseases showed the highest hazard ratio among 20–39 years old men.

Comorbid asthma is highly prevalent in CRS patients and is associated with severity and refractoriness of CRS^24^. A multinational survey study (n = 52,000) found that asthma was highly associated with CRS, especially in young people, which is consistent with our results^13^. A Taiwanese population-based study with the same setting as our Study I found an increased risk of CRSsNP in asthma patients, but the association with CRSwNP was not significant^25^. The HR for CRSsNP in our study was relatively higher than that for CRSwNP, although statistical significance was reached in both CRS phenotypes. This discrepancy may be explained by the smaller number of patients with CRSwNP compared to those with CRSsNP in both Korean and Taiwanese populations. Sinonasal tissues from Asian CRS patients were less eosinophilic in both CRSwNP and CRSsNP^26^. Furthermore, CRSwNP were also less eosinophilic in Chinese patients compared to European patients^27^. The regional difference of inflammatory patterns of CRS and asthma comorbidity might affect the low incidence of CRSwNP in both Asian national cohort studies. A study from the Canadian national cohort found that the presence of CRS resulted in an increased risk of developing asthma^28^. Another population-based study from Canada found CRS to be more frequent in asthma patients^29^.

Upper airway symptoms caused by infection or inflammation of the paranasal sinuses can hinder disease control or cause acute exacerbation of asthma^30^. Also, patient-reported severity of CRS is negatively associated with that of asthma control^31^. Surgical management of CRS, functional endoscopic sinus surgery (FESS), can reduce the severity of asthma, the frequency of attacks, and the number of medication use^32,33^. Regab *et al*. conducted a randomized study in CRS patients with comorbid asthma that assessed medical and surgical therapy of CRS, and found that both types of treatment had a positive effect on asthma control^34^. Among patients who underwent FESS due to recalcitrance to medical treatment, those who underwent operation earlier in the disease continuum had a decreased risk of new asthma diagnosis^10^.

Asthmatic CRS patients have terrible symptoms, including hyposmia and nasal blockage, compared to non-asthmatic CRS patients^35^. In addition, asthmatic CRS patients are known to be highly comorbid with nasal polyposis^5^. However, in this study, the prevalence of CRSsNP was higher than CRSwNP and the HR of CRSsNP was higher than that of CRSwNP. One of the possible reasons of the low incidence of CRSwNP is regional difference (Western versus Asia) as we mentioned above, and the other is possibility of the missing of J33 code in patients with CRSwNP in clinical setting. Usually clinicians give the patients a diagnostic code of J32 (chronic rhinosinusitis) and add J33 (nasal polyposis), if a patient has nasal polyps. There may be a chance that the J33 code is missing during the process. On the contrary, Marino *et al*. discovered that comorbid asthma did not impair sinus pneumatization and was not associated with the Lund-Mackay computed tomography (CT) score reflecting CRS disease severity^36^. In CRSwNP patients, comorbid asthma had no impact on tissue eosinophilia and CT score^37^. Further study is needed to determine whether asthma affects the development and progression of CRS.

Treatment with biologics is already popular in asthma patients and is increasingly being used for CRS, particularly CRSwNP patients^38,39^. The effectiveness of biologics treatment in asthmatic CRSwNP patients was proved by randomized controlled trial^40^. The findings of a bidirectional association between asthma and CRS could be used as evidence for biologics treatment in patients with CRS.

Although this study is the first to use a nationwide cohort to reveal the bidirectional association between asthma and CRS, it is not without limitations. First, the well-known risk factors of both diseases, such as smoking and obesity, could not analyzed due to the characteristics of the data. Second, although this result included a temporal relationship between the two diseases, the causal mechanism could not be elucidated. Third, CRSwNP might be underestimated due to missing J33 code. Lastly, a few cases who may have CRS history prior to 2012 could not be excluded in study 1, because there was no data before 2012.

In summary, our results show that CRS and asthma have a bidirectional influence on each other regardless of the presence of CRSwNP or CRSsNP. CRS increases the risk of asthma, and asthma increases the risk of CRS, especially in young men.

## Methods

### Study population and data collection

The ethics committee of the Institutional Review Board of Hallym University approved the use of these data (2017-I102). Written informed consent was waived by the Institutional Review Board of Hallym University. All methods of data management and analysis performed in accordance with the guidelines and regulations of the institutional ethic committee. We collected data from the Korean Health Insurance Review and Assessment Service-National Sample Cohort, as previously described in our studies^41–43^.

### Participant selection

Of 1,125,691 cases with 114,369,638 medical claim codes from 2002 to 2013, we included subjects diagnosed as asthma (ICD-10: J45) or status asthmaticus (J46). Among them, we included subjects who were diagnosed with asthma by a physician more than 2 times, and treated with asthma-related medications, including inhaled corticosteroid (ICS), ICSs combined with long-acting β2-agonists (LABAs), oral leukotriene antagonists (LTRAs), short-acting β2-agonists (SABAs), systemic LABAs, xanthine derivatives, and systemic corticosteroids (n = 230,764). The follow-up period was 12 years. The detailed method is described in the previous study^42,44^.

CRS was diagnosed using ICD-10 codes (J32). Among these, we selected the participants who had treatment more than 2 times due to CRS and had undergone head and neck CT (Claim codes: HA401-HA416, HA441-HA443, HA451-HA453, HA461-HA463, or HA471-HA473), as in our previous study^41^. Out of 1,125,691 cases, 5,177 participants were diagnosed as CRSwNP (J32 plus J33 for nasal polyposis), and 34,572 participants were diagnosed as CRSsNP (J32 only).

### Study I

The asthma patients were matched 1:1 with participants among this cohort who were not diagnosed with asthma (control I, n = 894,927). We matched variables between two groups, such as age, group, sex, income group, and region of residence. We sorted the control I group using another random number order and then selected from top to bottom to prevent selection bias. We set the index date as the date of the diagnosis of asthma, and we assumed that control I participants were involved at the same time as matched asthma participant^43^. Subjects who diagnosed CRS (J32) before the index date were excluded from the two groups. We excluded 5,963 participants from the asthma group because of the previous history of CRS and 20,682 participants due to insufficient matching. The average follow-up period from index date to last date (31st December 2013) or death date was almost similar in the asthma (86.1 months, SD = 42.9) and the control I group (85.6 months, SD = 43.2). After 1:1 matching, 204,119 patients with asthma and 204,119 control I participants were allocated (Fig. 1A). The occurrence of CRS was analyzed in the asthma and control I groups.

### Study II

We matched 1:4 of the CRS and control II group. The control II group (n = 1,085,942) was selected from the cohort who were not diagnosed with CRS from 2002 through 2013. Matching the variables (age, group, sex, income group, and region of residence) and setting the index date were performed same as Study I. In the CRS group, we excluded 8,744 participants who had previous history of asthma. There were no patients with CRS who could not sufficiently matched to the Control II. The mean follow-up time from index date to last date (31st December 2013) or death date was almost similar in the CRS group (87.5 months, SD = 41.2) and control II group (86.8 months, SD = 41.4). Therefore, 31,005 of CRS patients and 124,020 control II participants were included (Fig. 1B). We analyzed the occurrence of the asthma in the CRS and control II group. Also, we analyzed the occurrence of asthma in CRSwNP and their control, and CRSsNP and their control.

### Variables

The age of participants was grouped into 18 groups at five-year intervals (0–4, 5–9, though over 85 years old). The income was divided into 41 classes (one health aid class, 20 self-employment health insurance classes, and 20 employment health insurance classes), then we grouped into five classes [class 1 (the lowest income) to class 5(the highest income)]. The regions of residence were classified into urban (Seoul, Busan, Daegu, Incheon, Gwangju, Daejeon, and Ulsan) and rural (Gyeonggi, Gangwon, Chungcheongbuk, Chungcheongnam, Jeollabuk, Jeollanam, Gyeongsangbuk, Gyeongsangnam, and Jeju) areas.

COPD was defined as who were diagnosed J43 (emphysema) through J44 (Other chronic obstructive pulmonary disease), and treated with SABA, LABA, LAMA, and corticosteroid. Atopic dermatitis (L20) was defined that who was treated more than two time as a previous study^45^. Other 16 comorbidities except pulmonary disease were considered as a continuous variable [0 (no comorbidity) through 28 (multiple comorbidities)]^46^.

### Statistical analysis

The difference between disease and control group was analyzed by chi-squared test. A hazard ratio (HR) and 95% confidence interval of CRS or asthma was calculated using a Cox-proportional hazards model in the Study I and II, respectively. We stratified variables, such as age, sex, income, and region of residence were stratified, and adjusted atopic dermatitis (categorical variable), COPD (categorical variable), and CCI scores (continuous variable) in the adjusted model. We analyzed data using the Kaplan-Meier analysis and log-rank test.

We performed subgroup analysis according to the age and sex (<20 years old, 20–39 years old, 40–59 years old, and ≥60 years old; men and women). Additionally, we analyzed HRs according to nasal polyp histories. P < 0.05 was considered statistically significance. All analyses were performed using SAS version 9.4 (SAS Institute Inc., Cary, NC, USA) and SPSS version 22.0 (IBM, Armonk, NY, USA).

### Ethics approval and consent to participate

The ethics committee of the Institutional Review Board of Hallym University approved the use of these data (2017-I102) and written informed consent was waived.

## Supplementary information

Supplementary Information.

## Data Availability

Data are available for researchers who meet the criteria for access to confidential data.
