# Supplementary material for: Bidirectional association between asthma and chronic rhinosinusitis: Two longitudinal follow-up studies using a national sample cohort
Source: Sci Rep. 2020 Jun 12;10:9589. doi: 10.1038/s41598-020-66479-8 (PMC7293248; doi:10.1038/s41598-020-66479-8)
Supplement: Supplementary file 1 — Supplementary Information. [file 41598_2020_66479_MOESM1_ESM.docx]

Supplementary information

**Bidirectional association between asthma and chronic rhinosinusitis: Two longitudinal follow-up studies using a national sample cohort**

Gwanghui Ryu^1^, Chanyang Min^2,3^, Bumjung Park^4^, Hyo Geun Choi^4*^, and Ji-Hun Mo^5,6*^

^1^Department of Otorhinolaryngology-Head and Neck Surgery, Soonchunhyang University College of Medicine, Cheonan, Korea

^2^Hallym Data Science Laboratory, Hallym University College of Medicine, Anyang, Korea

^3^Graduate School of Public Health, Seoul National University, Seoul, Korea

^4^Department of Otorhinolaryngology-Head & Neck Surgery, Hallym University College of

Medicine, Anyang, Korea

^5^Department of Otorhinolaryngology, Dankook University College of Medicine, Cheonan, Korea

^6^Beckman Laser Institute Korea, Dankook University College of Medicine, Cheonan, Korea

**Corresponding authors:** Hyo Geun Choi^*^ and Ji-Hun Mo^*^

**Table S1** Distributions of chronic rhinosinusitis with/without nasal polyp in Study I (asthma and control I).

| Characteristics | | Study I | | |
| --- | --- | --- | --- | --- |
|  |  | Asthma | Control I | P-value |
| CRSwNP (n, %) | | 650 (0.3) | 343 (0.2) | <0.001* |
| CRSsNP (n, %) | | 7,393 (3.6) | 3,842 (1.9) | <0.001* |

* Chi-square test, Significance at P < 0.05

CRSsNP, chronic rhinosinusitis without nasal polyp; CRSwNP, chronic rhinosinusitis with nasal polyp

**Table S2** Distributions of asthma in Study II (chronic rhinosinusitis and control II).

| Characteristics | | | Study II | |
| --- | --- | --- | --- | --- |
|  | |  | Asthma (n, %) | P-value |
| CRSwNP (n = 20,705) | | |  | <0.001* |
|  | CRSwNP | | 7,51 (16.5) |  |
|  | Control II for CRSwNP | | 1,427 (7.9) |  |
| CRSsNP (n = 132,320) | | |  | <0.001* |
|  | CRSsNP | | 5,519 (20.9) |  |
|  | Control II for CRSsNP | | 11,304 (10.7) |  |

* Chi-square test, Significance at P < 0.05

CRSsNP, chronic rhinosinusitis without nasal polyp; CRSwNP, chronic rhinosinusitis with nasal polyp

**Table S3** General characteristics of patients with chronic rhinosinusitis in study I.

| Characteristics | | | Study I | | |
| --- | --- | --- | --- | --- | --- |
|  | |  | CRSwNP (n, %) | CRSsNP (n, %) | P-value |
| Age (years old) | | |  |  | <0.001* |
|  | 0-4 | | 73 (7.4) | 3,859 (34.4) |  |
|  | 5-9 | | 67 (6.8) | 997 (8.9) |  |
|  | 10-14 | | 35 (3.5) | 314 (2.8) |  |
|  | 15-19 | | 17 (1.7) | 151 (1.3) |  |
|  | 20-24 | | 18 (1.8) | 173 (1.5) |  |
|  | 25-29 | | 34 (3.4) | 294 (2.6) |  |
|  | 30-34 | | 61 (6.1) | 427 (3.8) |  |
|  | 35-39 | | 78 (7.9) | 452 (4.0) |  |
|  | 40-44 | | 108 (10.9) | 465 (4.1) |  |
|  | 45-49 | | 93 (9.4) | 647 (5.8) |  |
|  | 50-54 | | 95 (9.6) | 713 (6.4) |  |
|  | 55-59 | | 96 (9.7) | 671 (6.0) |  |
|  | 60-64 | | 86 (8.7) | 711 (6.3) |  |
|  | 65-69 | | 70 (7.1) | 634 (5.6) |  |
|  | 70-74 | | 37 (3.7) | 433 (3.9) |  |
|  | 75-79 | | 17 (1.7) | 207 (1.8) |  |
|  | 80-84 | | 8 (0.8) | 66 (0.6) |  |
|  | 85+ | | 0 (0.0) | 21 (0.2) |  |
| Sex | | |  |  | 0.010* |
|  | Male | | 528 (53.2) | 5,494 (48.9) |  |
|  | Female | | 465 (46.8) | 5,741 (51.1) |  |
| Income | | |  |  | 0.007* |
|  | 1 (lowest) | | 135 (13.6) | 1,167 (10.4) |  |
|  | 2 | | 130 (13.1) | 1,441 (12.8) |  |
|  | 3 | | 180 (18.1) | 2,220 (19.8) |  |
|  | 4 | | 245 (24.7) | 3,133 (27.9) |  |
|  | 5 (highest) | | 303 (30.5) | 3,274 (29.1) |  |
| Region of residence | | | |  | 0.148 |
|  | Urban | | 435 (43.8) | 5,190 (46.2) |  |
|  | Rural | | 558 (56.2) | 6,045 (53.8) |  |
| Atopic dermatitis | | | 84 (8.5) | 2,577 (22.9) | <0.001* |
| COPD | | | 220 (22.2) | 1,292 (11.5) | <0.001* |
| CCI (score)† | | |  |  | <0.001* |
|  | 0 | | 283 (28.5) | 5,515 (49.1) |  |
|  | 1 | | 175 (17.6) | 1,370 (12.2) |  |
|  | ≥ 2 | | 535 (53.9) | 4,350 (38.7) |  |
| Asthma | | | 650 (65.5) | 7,393 (65.8) | 0.826 |

* Chi-square test, Significance at P < 0.05

CRSsNP, chronic rhinosinusitis without nasal polyp; CRSwNP, chronic rhinosinusitis with nasal polyp; COPD, chronic obstructive pulmonary disease

† Charlson Comorbidity Index was calculated without pulmonary disease.

**Table S4** General characteristics of patients with chronic rhinosinusitis in study II.

| Characteristics | | Study II | | |
| --- | --- | --- | --- | --- |
|  |  | CRSwNP (n, %) | CRSsNP (n, %) | P-value |
| Age (years old) | |  |  | <0.001* |
|  | 0-4 | 18 (0.4) | 2,127 (8.0) |  |
|  | 5-9 | 123 (2.7) | 1,856 (7.0) |  |
|  | 10-14 | 234 (5.2) | 1,512 (5.7) |  |
|  | 15-19 | 248 (5.5) | 1,394 (5.3) |  |
|  | 20-24 | 240 (5.3) | 1,240 (4.7) |  |
|  | 25-29 | 315 (6.9) | 1,635 (6.2) |  |
|  | 30-34 | 433 (9.5) | 1,985 (7.5) |  |
|  | 35-39 | 464 (10.2) | 2,059 (7.8) |  |
|  | 40-44 | 458 (10.1) | 2,042 (7.7) |  |
|  | 45-49 | 494 (10.9) | 2,143 (8.1) |  |
|  | 50-54 | 432 (9.5) | 2,108 (8.0) |  |
|  | 55-59 | 392 (8.6) | 1,878 (7.1) |  |
|  | 60-64 | 287 (6.3) | 1,544 (5.8) |  |
|  | 65-69 | 200 (4.4) | 1,355 (5.1) |  |
|  | 70-74 | 127 (2.8) | 897 (3.4) |  |
|  | 75-79 | 53 (1.2) | 450 (1.7) |  |
|  | 80-84 | 16 (0.4) | 171 (0.6) |  |
|  | 85+ | 7 (0.2) | 68 (0.3) |  |
| Sex | |  |  | 1.000 |
|  | Male | 2,914 (64.2) | 13,105 (49.5) |  |
|  | Female | 1,627 (35.8) | 13,359 (50.5) |  |
| Income | |  |  | 0.053 |
|  | 1 (lowest) | 551 (12.5) | 3,308 (12.5) |  |
|  | 2 | 648 (14.3) | 3,417 (12.9) |  |
|  | 3 | 819 (18.0) | 4,682 (17.7) |  |
|  | 4 | 1,127 (24.8) | 6,497 (24.6) |  |
|  | 5 (highest) | 1,396 (30.7) | 8,560 (32.3) |  |
| Region of residence | |  |  |  |
|  | Urban | 2,094 (46.1) | 12,994 (49.1) |  |
|  | Rural | 2,447 (53.9) | 13,470 (50.9) |  |
| Atopic dermatitis | | 249 (5.5) | 3,094 (11.7) | <0.001* |
| COPD | | 349 (7.7) | 1,770 (6.7) | 0.014 |
| CCI (score)† | |  |  |  |
| 0 | | 1,400 (30.8) | 10,482 (39.6) |  |
| 1 | | 1,046 (23.0) | 4,195 (15.9) |  |
| ≥ 2 | | 2,095 (46.1) | 11,787 (44.5) |  |
| Asthma | | 751 (16.5) | 5,519 (20.9) | <0.001* |

* Chi-square test, Significance at P < 0.05

CRSsNP, chronic rhinosinusitis without nasal polyp; CRSwNP, chronic rhinosinusitis with nasal polyp; COPD, chronic obstructive pulmonary disease

† Charlson Comorbidity Index was calculated without pulmonary disease.
